# Association between mortality and cardiovascular diseases in the vulnerable Mexican population: A cross-sectional retrospective study of the COVID-19 pandemic

**DOI:** 10.3389/fpubh.2022.1008565

**Published:** 2022-11-10

**Authors:** Gerardo R. Padilla-Rivas, Juan Luis Delgado-Gallegos, Gerardo Garza-Treviño, Kame A. Galan-Huerta, Zuca G-Buentello, Jorge A. Roacho-Pérez, Michelle Giovana Santoyo-Suarez, Hector Franco-Villareal, Ahidée Leyva-Lopez, Ana E. Estrada-Rodriguez, Jorge E. Moreno-Cuevas, Javier Ramos-Jimenez, Ana M. Rivas-Estrilla, Elsa N. Garza-Treviño, Jose Francisco Islas

**Affiliations:** ^1^Departamento de Bioquímica y Medicina Molecular, Facultad de Medicina, Universidad Autónoma de Nuevo León, Monterrey, Nuevo León, México; ^2^Althian Clinical Research, Monterrey, Nuevo León, México; ^3^Centro de Investigación en Salud Poblacional Instituto Nacional de Salud Pública, Cuernavaca, Morelos, México; ^4^Departamento de Ciencias Básicas, Universidad de Monterrey, San Pedro Garza García, México

**Keywords:** cardiovascular diseases, Mexico COVID-19, México metropolitan area, COVID-CVDs, COVID mortality in Mexico

## Abstract

Cardiovascular diseases (CVDs) continue to be the leading cause of death worldwide. Over the past couple of years and with the surge of the COVID-19 pandemic, mortality from CVDs has been slightly overshadowed by those due to COVID-19, although it was during the peak of the pandemic. In the present study, patients with CVDs (CVDs; *n* = 41,883) were analyzed to determine which comorbidities had the largest impact on overall patient mortality due to their association with both diseases (*n* = 3,637). Obesity, hypertension, and diabetes worsen health in patients diagnosed positive for COVID-19. Hence, they were included in the overview of all patients with CVD. Our findings showed that 1,697 deaths were attributable to diabetes (*p* < 0.001) and 987 deaths to obesity (*p* < 0.001). Lastly, 2,499 deaths were attributable to hypertension (*p* < 0.001). Using logistic regression modeling, we found that diabetes (OR: 1.744, *p* < 0.001) and hypertension (OR: 2.179, *p* < 0.001) significantly affected the mortality rate of patients. Hence, having a CVD diagnosis, with hypertension and/or diabetes, seems to increase the likelihood of complications, leading to death in patients diagnosed positive for COVID-19.

## Introduction

The Metropolitan Area of the Valley of Mexico is the largest metropolitan area in North America; it is the country's most important economic, political, and social hub. It houses over 22 million people, roughly 20% of the country's population (1). Given the size and importance of this area, the federal government has devoted much of the national healthcare system's resources to tackling many public health issues. The area is host to many Tier-1 national healthcare facilities in the nation, hence promoting health-related tourism (2–5).

One of the biggest challenges the world has faced since the last century has been the increase in mortality from cardiovascular diseases (CVDs) (6, 7), and Mexico has been no exception, particularly since the second half of the past century (8). To make things even more difficult, since the beginning of the coronavirus disease 2019 (COVID-19) pandemic, Mexico has been one of the worst hit countries in the world, with an observed case-fatality ratio of 9% at its peak (9, 10). Fortunately, as face mask mandates were implemented and new effective vaccines became available (11), this percentage dropped to around 5.5%. Unfortunately, the case-fatality ratio in Mexico is still double than that of Indonesia (2.6%), with about 253.8 deaths per 100,000 population (12). Unsurprisingly, CVDs and their associated comorbidities, such as hypertension, obesity, and diabetes, were identified early in the pandemic as being associated with increased mortality (13, 14). One reason behind CVD's high mortality during the COVID-19 pandemic is that hypertension, obesity, and diabetes upregulate highly inflammatory states in the individual and, as a result, in combination with COVID-19, activate hyper-inflammatory reactions, resulting in a potential dysregulation of the immune system (15).

During a severe acute respiratory syndrome coronavirus 2 (SARS-CoV-2) infection, which gives rise to COVID-19, the angiotensin-converting-enzyme 2 (ACE2) receptor, expressed in many human tissues, including the lungs, the heart, the kidneys, the liver, and the nervous system, plays a crucial role in mediating viral entry (16). Lung-specific ACE2 catalyzes the conversion of angiotensin I to angiotensin II (17, 18), which then activates the AT1 receptor to trigger a proinflammatory cascade characterized by inflammation, vasoconstriction, aldosterone production, and an increase in oxidative stress (19). When this proinflammatory cascade is triggered, it might lead to endothelial dysfunction. Comorbidities such as obesity, diabetes, and hypertension increase the severity of CVDs and their associated comorbidities (15, 20).

In the present study, we chose to analyze COVID-19 pandemic data from early 2020 through the first trimester of 2022; we obtained them from the healthcare system in the Mexico metropolitan region. We selected patients diagnosed with CVD and paid particular attention to the vulnerable or high-risk populations, such as those affected by comorbidities such as obesity, diabetes, and hypertension, as well as those infected with COVID-19.

## Materials and methods

Publicly available data were obtained from the Ministry of Health of Mexico City, which included >5.5 million total entries from 2020 to the first trimester (march) of 2022 (21). This system was used to register COVID-19 diagnoses for the population of Mexico City, including private and public health services. Once we obtained the data, we sorted through all individuals deemed to have CVDs. Our final count was 41,882 individuals. Next, individual frequencies were calculated for gender (female/male), age groups, COVID-19 diagnosis (test results: positive/negative/unknown-untested), medical attention (hospitalized/ambulatory), obesity, diabetes, hypertension (yes/no/not reported), death (yes/no), and year (2020/2021/2022).

### Deaths by medical attention and diagnosis

Death was taken as the main variable for all patients with CVD. First, we recorded COVID-19-associated fatalities by year. Next, we compared death, the diagnosis of COVID-19, and the type of medical care.

### COVID-19 and death results by comorbidities

All patients with CVD were then classified according to COVID-19 diagnoses and subsequently classified according to their comorbidities: hypertension/diabetes, hypertension/obesity, and obesity/diabetes. As with comorbidities, we only calculated the subgroup of deceased individuals.

### Age groups, gender, and results

For all patients with CVD, we divided each comorbidity, type of medical attention, mortality, and COVID-19 diagnosis by age, group, and gender.

### Chi-squared correlations and binary logistic regression

For all patients with CVD, we calculated statistical correlations between each comorbidity and each group's mortality. Next, we built a binary logistic regression model to determine the influence each comorbidity and a positive COVID-19 diagnosis would have on the mortality of patients with CVD. All statistical analyses, including binary logistic regression, Pearson's chi-squared, and an R ratio of 0.05, were performed using IBM SPSS Statistics for Windows (version 23.0) (IBM Corp., Armonk, NY, USA). A parallel set was further made in RStudio v. 1.3.1093 with R v. 4.0.3. We used the following packages: reshape2 v. 1.4.4, tidyverse v. 1.3.0, and ggforce v. 0.3.3. These packages can be downloaded from CRAN (https://cran.r-project.org/).

## Results

The Ministry of Health of Mexico City released figures showing that over 5.5 million patients were registered from the beginning of 2020 to the first trimester of 2022 (March). These data were sorted for the specific subgroup of patients diagnosed with CVDs. The latter group consisted of 41,882 entries with 3,697 patient-reported deaths. Of the total of patients diagnosed with CVD, female entries represented 51.8% of the total entries. Considering the demographic factor of age, the number of participants was higest between the ages of 51 and 60 (20.8%), followed by the ages of 61–70 (*n* = 8,147, 19.5%). The total number of patients diagnosed positive for COVID-19 (*n* = 15,363) was 36.7%. Additionally, ambulatory patients (*n* = 32,108) represented 76.3%. Regarding comorbidities, 19,405 patients (46.3%) were positive for hypertension, 11,879 for (28.4%) diabetes, and 9,612 (23%) for obesity. Finally, while 2022 is still ongoing, during the first 2 years, there were a total of 34,573 patients, with 16,565 patients in 2020 (48%) and 18,008 patients in 2021 (52%). Data are shown in Table 1.

**Table 1:** Profile for patients diagnosed with CVDs (*n* = 41,882).

	***n***	**%**
**Gender**		
Women	21,701	51.8
Men	20,181	48.2
**Age groups**		
0–17	2,029	4.8
18–30	3,797	9.1
31–40	3,956	9.4
41–50	6,205	14.8
51–60	8,691	20.8
61–70	8,147	19.5
71–80	5,779	13.8
81 and more	3,278	7.8
**COVID-19**		
Positive	15,363	36.7
Negative	24,659	58.9
Unknown/untested	1,860	4.5
**Type of medical attention**		
Ambulatory	32,108	76.3
Hospitalized	9,974	23.7
**Hypertension**		
Yes	19,405	46.3
No	22,477	53.7
**Diabetic**		
Yes	11,879	28.4
No	30,003	71.6
**Obesity**		
Yes	9,612	23.0
No	32,270	77.0
**Death**		
Yes	3,637	8.7
No	38,246	91.3
**Year**		
2020	16,565	39.6
2021	18,008	43.0
2022^*^	7,309	17.5

* Data for the first trimester of 2022.

We then tallied deaths among CVD patients diagnosed positive for COVID-19 (by year) and deaths among patients with CVD who received a specific kind of medical care (See Tables 2A,B). Next, we showed the relationship between the variables in Table 2C. In Table 2A, we can observe that the yearly average of having a COVID-19-positive diagnosis is approximately 73%. Another interesting observation is hospitalization and its relation to mortality. Of the total of 3,637 deaths, only 255 ambulatory patients died (Table 2B). When we further analyzed mortality, type of medical attention, and COVID-19 diagnosis, we observed that the highest mortality was in hospitalized patients, with a total of 2,373 recorded deaths, plus an additional 790 deaths with no COVID-19 diagnosis (Table 2C).

**Table 2A:** COVID-19 diagnosis (deaths by year).

**Year**	**Positive**		**Negative**		**Unknown/untested**		**Total**
	***N***	**%**	***N***	**%**	***n***	**%**	
2020	1,358	67	494	24.40	175	8.60	2,027
2021	1,009	72.70	311	22.40	68	4.90	1,388
2022^*^	174	78.40	43	19.40	5	2.30	222

* Data for the first trimester of 2022.

**Table 2B:** Type of medical attention (deaths).

**Death**	**Yes**		**No**		**Total**
	***n***	**%**	***N***	**%**	
Ambulatory	255	0.80%	31,853	99.2	32,108
Hospitalized	3,382	34.60%	6,392	65.40%	9,774
Total	3,637		38,245		41,882

**Table 2C:** COVID-19 diagnosis/type of medical attention (deaths).

**COVID-19**	**Patient type**	**Death**	***n***	**%**	**Total**
Positive	Hospitalized	Yes	2,373	49.68	4,777
		No	2,404	50.32	
	Ambulatory	Yes	168	1.59	10,586
		Np	10,418	98.41	
Negative	Hospitalized	Yes	790	18.41	4,291
		No	3,501	81.59	
	Ambulatory	Yes	58	0.28	20,368
		No	20,310	99.72	
Unknown/untested	All	Yes	248	13.30	1,860
		No	1,612	86.67	
				Total	41,882

This study aimed to analyze the impact of CVD and comorbidities on COVID-19-related mortality. First, we indentified 949 cases related to mortality due to COVID-19-positive diagnosis with both hypertension and diabetes comorbidities (regardless of obesity diagnosis). Interestingly, diabetes alone resulted in only 244 deaths, while no comorbidity additional to COVID-19 diagnosis showed 518 deaths. Moreover, hypertension seems to be a major contributor to death, as 830 cases were attributable to hypertension, as shown in Table 3A.

**Table 3A:** Death related to COVID-19 (hypertension/diabetes).

**COVID-19**	**Hypertension**	**Diabetes**	***n***	**%**	**Total**
Positive	Yes	Yes	949	53.34	1,779
		No	830	46.66	
	No	Yes	244	32.02	762
		No	518	67.98	
Negative	Yes	Yes	310	55.66	557
		No	247	44.34	
	No	Yes	76	26.12	291
		No	215	73.88	
Unknown/untested	All	Yes	118	47.58	248
		No	130	52.42	
				Total	3,637

Next, we continued linking deaths to hypertension as the primary comorbidity. Then, we started linking obesity along with hypertension (without accounting for diabetes). Mortality from COVID-19-positive diagnosis and both conditions resulted in 578 cases of mortality. Within this group, hypertension alone was responsible for 1,201 additional deaths, while obesity accounted for 158 deaths, as shown in Table 3B.

**Table 3B:** Death related to COVID-19 (hypertension/obesity).

**COVID-19**	**Hypertension**	**Obesity**	***n***	**%**	**Total**
Positive	Yes	Yes	578	32.49	1,779
		No	1,201	67.51	
	No	Yes	158	20.73	762
		No	604	79.27	
Negative	Yes	Yes	164	29.44	557
		No	393	70.56	
	No	Yes	46	15.81	291
		No	245	84.19	
Unknown/	All	Yes	41	16.53	248
untested		No	207	83.47	
				Total	3,637

Additionally, we analyzed the impact of CVD and COVID-19 diagnoses on diabetes and obesity. Table 3C indicates that having both comorbidities resulted in 736 deaths, whereas diabetes alone contributed to 815 additional deaths, and obesity alone led to 358 deaths.

**Table 3C:** Death related to COVID-19 (obesity/diabetes).

**COVID-19**	**Obesity**	**Diabetes**	***n***	**%**	**Total**
Positive	Yes	Yes	378	51.36	736
		No	358	48.64	
	No	Yes	815	45.15	1,805
		No	990	54.85	
Negative	Yes	Yes	118	56.19	210
		No	92	43.81	
	No	Yes	268	42.01	638
		No	370	57.99	
Unknown/untested	All	Yes	118	47.58	248
		No	130	52.42	
				Total	3,637

Lastly, we studied the contributions of all three comorbidities in relation to death and COVID-19 diagnosis. We observed that diabetes alone contributed to a total of 184 deaths. Next, the highest contributor to death was hypertension, resulting in 570 cases, and the least amount of individual contribution was attributed to obesity, as it only resulted in 98 deaths, as shown in Table 3D.

**Table 3D:** Total deaths related to COVID-19 (diabetes/hypertension/obesity).

**COVID-19**	**Diabetes**	**Hypertension**	**Obesity**	***n***	**%**	**Total**
Positive	Positive	Yes	Yes	318	33.51	949
			No	631	66.49	
		No	Yes	60	24.59	244
			No	184	75.41	
	Negative	Yes	Yes	260	31.33	830
			No	570	68.67	
		No	Yes	98	18.92	518
			No	420	81.08	
Negative	Positive	Yes	Yes	101	32.58	310
			No	209	67.42	
		No	Yes	17	22.37	76
			No	59	77.63	
	Negative	Yes	Yes	63	25.51	247
			No	184	74.49	
		No	Yes	29	13.49	215
			No	186	86.51	
Unknown/untested	All	All	Yes	38	15.32	248
			No	210	84.68	
					Total	3,637

Additional analysis, including deaths resulting from patients diagnosed negative for COVID-19, is shown in Tables 3A–D.

Next, we analyzed total deaths in patients with CVD and COVID-19 and all three comorbidities by year (Supplementary Table 1). The results showed that, in 2020, the total number of deaths recorded was 2,027, of which the highest number, 315, was due to COVID-19, diabetes, and hypertension. Remarkably, the subsequent high incidence was 312 for COVID-19 and hypertension. The number of deaths in 2021 decreased to 1,388, with a decrease by nearly 31% from the previous year. The greatest incidence observed was 262, seen in patients who tested positive for COVID-19 and those with diabetes and hypertension. Additionally, the second greatest occurrence rate (*n* = 214) was in hypertension patients who diagnosed positive COVID-19.

To further show the influence of each comorbidity and a positive diagnosis for COVID-19, we performed a binary logistic regression. Our results showed no statistical correlation between obesity and death (*p* = 0.755). Meanwhile, a positive COVID-19 diagnosis showed the overall strongest correlation (*p* < 0.01, OR: 4.377), followed by hypertension (*p* < 0.01, OR: 2.179) and diabetes (*p* < 0.001, OR: 1.744), Table 4.

**Table 4:** Binary logistic regression analysis on death and comorbidities.

	***B***	**SE**	**Wald**	**Gl**	***p*-Values**	**OR**	**95% CI for OR**	
							**Inferior**	**Superior**
Diabetes	0.556	0.038	208.724	1	<0.001	1.744	1.617	1.880
Hypertension	0.779	0.040	369.788	1	<0.001	2.179	2.013	2.359
Obesity	−0.013	0.041	0.097	1	0.755	0.987	0.910	1.070
COVID-19	1.476	0.038	1,498.193	1	<0.001	4.377	4.062	4.717

Model summary: −2 log-likelihood = 22,019.540, Cox and Snell R square = 0.063, and Nagelkerke R square = 0.140.

**Figure 1:**
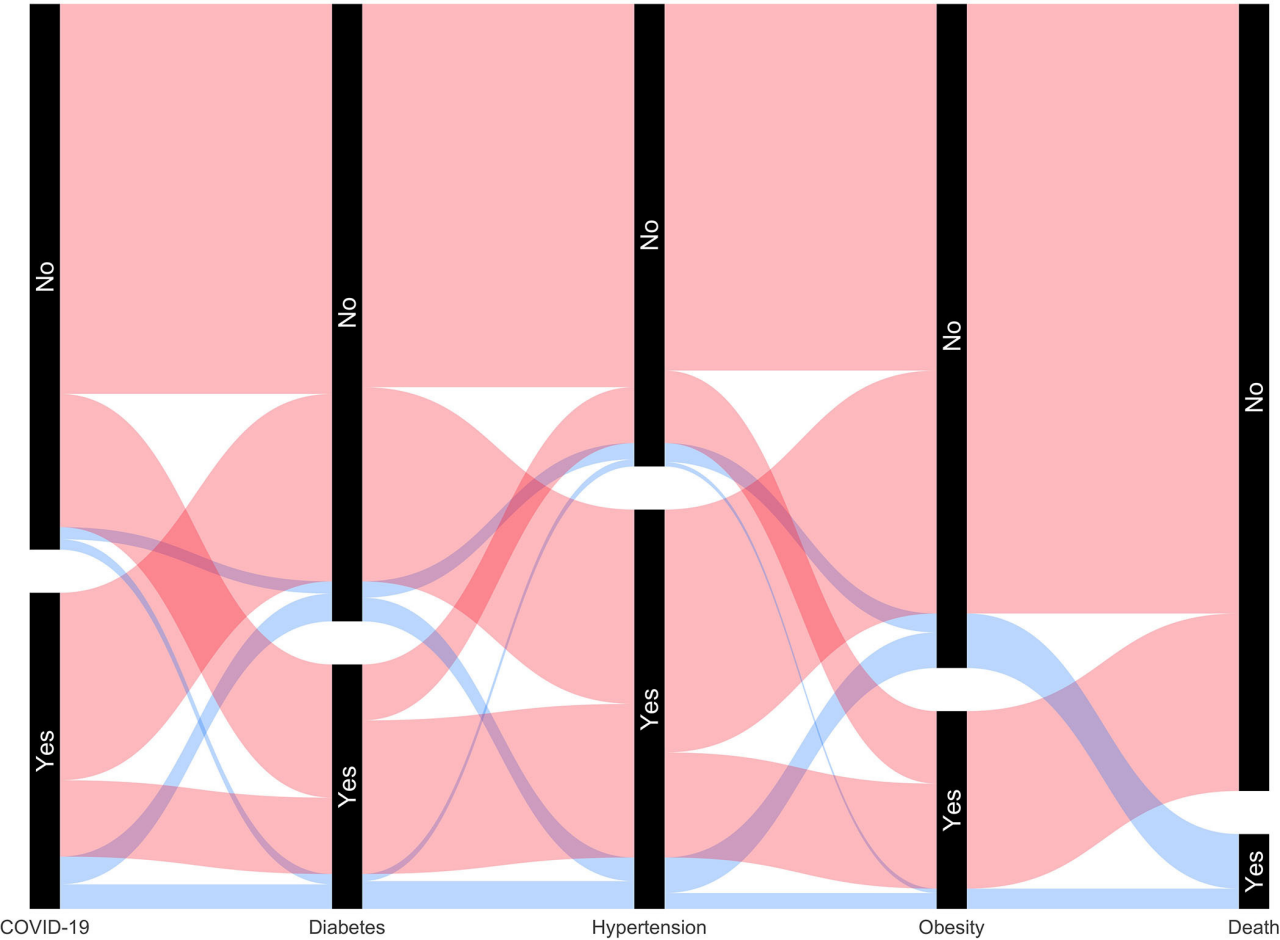
Parallel set visualization of the total number of patients with COVID-19 and other comorbidities, including death.

**Figure 2:**
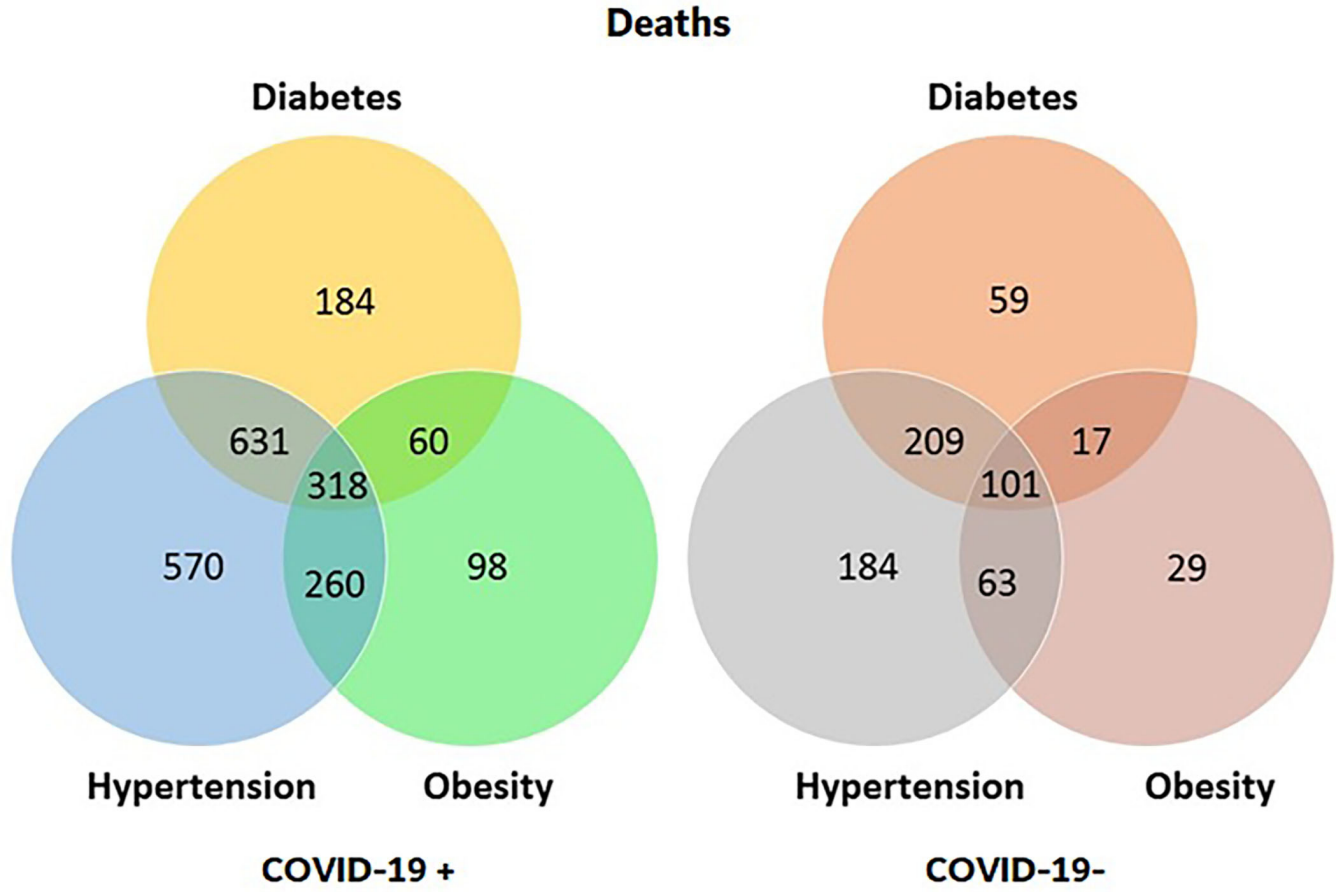
Diagrams of total deaths for patients with CVD, divided by COVID-19 diagnosis, with values of comorbidities either individually or combined.

Finally, we correlated individual comorbidities to death, demonstrating high correlations as all three comorbidities had *p*-values of < 0.01 (See Supplementary Table 2). Parallel set visualization of the data is shown in Figure 1. Figure 2 is a Venn diagram depicting the total number of deaths due to each comorbidity separately and together.

## Discussion

A recent statement by the American Heart Association mentioned that CVDs continue to be one of the most frequent causes of mortality worldwide (22). In the US alone, reports showed that CVDs account for the death of 1 person every 36 s and one in three deaths in women (23, 24). Given the recent COVID-19 pandemic, it is likely that deaths attributed to COVID-19 during the highest peaks of the pandemic could have overshadowed the mortality from CVDs (including patients with related comorbidities). Fortunately, novel vaccines are being developed and distributed all over the world. Vaccine acceptance, safe distancing practices, and overall better public conscientization have significantly diminished COVID-19 mortality (9, 11, 25–28). For Mexico, this has eventually resulted in a drop from the 9% COVID-19 case-fatality ratio, at its highest, to just above 5% (12).

In this study, we reviewed data from people living in the metropolitan area of the Mexico City who were diagnosed with cardiovascular diseases and treated at either an outpatient clinic or a hospital. About 20% of Mexico's population lives in the greater Mexico City area, which is also home to some of the country's top reference hospitals. Because of this, many people go to Mexico for medical care (2–5). A total of 41,882 people were diagnosed for CVD over a period of 2 years and three months, with 51.8% of those people being women. Of the 3,637 deaths recorded, 38.2% of those people were women (Table 2).

Over the course of the COVID-19 pandemic, several studies showed that patients with CVD are at higher risk of death, particularly when these include comorbidities such as hypertension, obesity, and diabetes, making them particularly vulnerable groups (13, 29–31). In a recent study, which included 46,321 COVID-19-positive hospitalized individuals (with CVDs), the mortality rates were found to be four times higher in patients with comorbidities such as diabetes (OR: 2.41 *p* < 0.001) and hypertension (OR: 2.60 *p* < 0.001) (32). Our study focused on patients with CVDs, finding similar trends for both diabetes (OR: 1.744, *p* < 0.001) and hypertension (OR: 2.179, *p* < 0.001), as well as observing that 92% of deaths (*n* = 3.382, Table 2B) were recorded in hospitalized patients, of which 2,393 were diagnosed positive for COVID-19. When we further performed chi-squared correlations of deaths for individual comorbidity contributions (Supplementary Table 2), we found that, of the 3,637 deaths, 987 deaths were attributed directly to obesity (*p* < 0.001), while 2,499 were attributed to hypertension (*p* < 0.001) and 1,697 were due to diabetes (*p* < 0.001). In our study, the total number of reported hospitalized patients (regardless of condition) was 9,774, suggesting the death of 34.6% of hospitalized patients.

An interesting observation was made with the total number of deaths (Tables 2A, 3, and Supplementary Table 1): a steady drop over time from 2020 to 2021, a 31% decrease. Overall, data showed that COVID-19 vaccination has greatly reduced the number of cases from that during the COVID-19 pandemic. In addition, it is important to note that, since late December 2020, the Mexican government has been making concerted efforts to vaccinate the population, starting with those most at risk. Although the availability of vaccines has helped assuage concerns worldwide, they are not a panacea by themselves. By late November 2021, official government reports showed that nearly 58% of the population in Mexico had received at least one dose of vaccination (33). To stop the spread of COVID-19, it is essential to use PPE and maintain social distancing. The relaxation of these restrictions has led to the emergence of COVID-19 variations (34). Recently, a fresh wave of COVID-19 infections has appeared in the northeastern United States. New York and Massachusetts, among others, are on high alert as they reevaluate their face mask laws (35). A recent report revealed that, in several countries where COVID-19 restrictions have been relaxed, there had been subsequent increases not only in overall cases but also in hospitalizations and deaths. However, the severity of the case is still under investigation (36).

Finally, while patients with CVD are an at-risk population, particularly when additional comorbidities are present, it is important to ponder the opposite question: Are post-COVID-19 patients at risk for CVDs? While this was not the focus of the current study, a recent study by Al-Aly et al. showed that COVID-19 survivors, regardless of age or race, had an increased risk of developing CVD, highlighting the need for physicians to include COVID-19 diagnosis as part of a history of CVDs (37). Interestingly, inflammatory heart diseases were independently confirmed for a group of professional athletes. Just over half of the tested athletes have had a previous COVID-19 diagnosis. Of these individuals, 45 showed either abnormal ECGs or elevated troponins, which caused concern, restricting their play. We should note that long-term follow-up is ongoing (38). In another study, about half of the 1,216 participants from over 69 countries (aged 52–79) who also had a history of COVID-19 showed abnormal electrocardiography. Their results showed left and right ventricular abnormalities in 479 (39%) and 397 (33%) patients, myocardial infarction in 36 (3%), myocarditis in 35 (3%), and takotsubo cardiomyopathy in 19 of them (2%) (39).

Having a CVD diagnosis, particularly with comorbidities, seems to increase the likelihood of complications leading to death in patients tested positive for COVID-19. Meanwhile, having a COVID-19 diagnosis seems to increase the risk of developing CVDs in the long run. While both conditions are still under investigation, individuals should strive to pursue healthy lifestyles to reduce the potential of developing CVDs and take preventive measures such as using face masks and vaccines to minimize the possibility of getting COVID-19. These perspectives are essential to enhance the quality of life in individuals and reduce any potential complications one could develop from these or other related diseases.

## Limitations

Understanding vulnerable populations is a multi-complex variable problem. Currently, we are focused on considering the particular population of patients with CVD and their relation to mortality based on three of the most important comorbidities (diabetes, hypertension, and obesity) as determined by several studies, as well as the influence of COVID-19. Further analysis with other comorbidities, pharmacological intake, social, economic, educational, and stress-related factors (including religious beliefs), and the COVID-19 variant should be performed to help model risk factors in these and other vulnerable populations, as uncertainty for this pandemic and any future one is massive.

## Conclusions

When the COVID-19 epidemic hit, Mexico was among the worst hit countries. In particular, Mexico City was among the most affected cities in the country. Our findings suggest that obesity does not seem to contribute significantly to CVD and COVID-19-related mortality; both diabetes and hypertension seem to be key contributors to mortality from CVD when confronted with a positive COVID-19 diagnosis. It is essential for public health officials and policymakers to enhance medical follow-up strategies for these particularly vulnerable groups, as prompt diagnosis and pharmacological and non-pharmacological treatment for patients may act as protective factors against COVID-19. Additionally, evidence showed that COVID-19 vaccination plays a crucial role in the mortality reduction of these particular populations. Therefore, vaccination strategies should prioritize these populations to reduce mortality, as potential waves of COVID-19 (and its variations) may continue to rise in the years to come.

## Data availability statement

Publicly available datasets were analyzed in this study. This data can be found at: https://datos.cdmx.gob.mx/group/covid-19.

## Author contributions

GP-R, JD-G, EG-T, and JI conceptualized and supervised the study and contributed to the overall design of the survey experiment, analysis, and interpretation of the data. JD-G and JI wrote the first draft of the manuscript. MS-S, GG-T, KG-H, and ZG-B contributed to the overall design of the experiment. JAR-P, HF-V, JM-C, and AE-R contributed to the analysis and statistical interpretation of the data. AL-L, AR-E, and JR-J contributed to the discussion. All authors had access to all the data in this study and bore final responsibility for the decision to submit it for publication.

## Conflict of interest

The authors declare that the research was conducted in the absence of any commercial or financial relationships that could be construed as a potential conflict of interest.

## Publisher's note

All claims expressed in this article are solely those of the authors and do not necessarily represent those of their affiliated organizations, or those of the publisher, the editors and the reviewers. Any product that may be evaluated in this article, or claim that may be made by its manufacturer, is not guaranteed or endorsed by the publisher.
